# Para-chloro-2-[^18^F]fluoroethyl-etomidate: A promising new PET radiotracer for adrenocortical imaging

**DOI:** 10.7150/ijms.51206

**Published:** 2021-03-21

**Authors:** Isabella Silins, Anders Sundin, Patrik Nordeman, Mahabuba Jahan, Sergio Estrada, Azita Monazzam, Mark Lubberink, Franklin Aigbirhio, Per Hellman, Gunnar Antoni

**Affiliations:** 1 Department of Surgical Sciences, Uppsala University.; 2 Medicinal Chemistry and Uppsala University.; 3 Medical Sciences at Uppsala University.; 4 Wolfson Brain Imaging Centre, University of Cambridge.

**Keywords:** 18F-CETO, Adrenal masses, Adrenal tracer, Positron emission tomography, Primary aldosteronism

## Abstract

**Introduction:** [^11^C]Metomidate ([^11^C]MTO), the methyl ester analogue of etomidate, was developed as a positron emission tomography (PET) radiotracer for adrenocortical tumours and has also been suggested for imaging in primary aldosteronism (PA). A disadvantage of [^11^C]MTO is the rather high non-specific binding in the liver, which impacts both visualization and quantification of the uptake in the right adrenal gland. Furthermore, the short 20-minute half-life of carbon-11 is a logistic challenge in the clinical setting.

**Objectives:** The aim of this study was to further evaluate the previously published fluorine-18 (T_1/2_=109.5 min) etomidate analogue, para-chloro-2-[^18^F]fluoroethyl etomidate; [^18^F]CETO, as an adrenal PET tracer.

**Methods:** *In vitro* experiments included autoradiography on human and cynomolgus monkey (non-human primate, NHP) tissues and binding studies on adrenal tissue from NHPs. *In vivo* studies with [^18^F]CETO in mice, rats and NHP, using PET and CT/MRI, assessed biodistribution and binding specificity in comparison to [^11^C]MTO.

**Results:** The binding of [^18^F]CETO in the normal adrenal cortex, as well as in human adrenocortical adenomas and adrenocortical carcinomas, was shown to be specific, both *in vitro* (in humans) and *in vivo* (in rats and NHP) with an *in vitro* K_d_ of 0.66 nM. Non-specific uptake of [^18^F]CETO in NHP liver was found to be low compared to that of [^11^C]MTO.

**Conclusions:** High specificity of [^18^F]CETO to the adrenal cortex was demonstrated, with *in vivo* binding properties qualitatively surpassing those of [^11^C]MTO. Non-specific binding to the liver was significantly lower than that of [^11^C]MTO. [^18^F]CETO is a promising new PET tracer for imaging of adrenocortical disease and should be evaluated further in humans.

## Introduction

Adrenocortical imaging by positron emission tomography (PET) with [^11^C]metomidate ([^11^C]MTO) is currently performed in specialized centres. [^11^C]MTO is specific for adrenocortical cells by binding to the enzymes encoded for by the genes *CYP11B1* (11β-hydroxylase) and *CYP11B2* (aldosterone synthase). [^11^C]MTO-PET has been helpful in characterizing equivocal retroperitoneal tumours, diagnosis of adrenocortical carcinoma (ACC) and in visualizing metastases in ACC staging and in recurrent disease 1-3. A recently proposed additional imaging application is for lateralization of primary aldosteronism (PA) in order to make invasive adrenal vein sampling redundant 4. With PA being a treatable cause of hypertension, representing of 3-13% of this widespread global health disorder, there is a major need for an accurate diagnostic technique 5-7.

The use of [^11^C]MTO PET/CT is somewhat hampered by considerable accumulation in the liver, which, because of its close proximity to the right adrenal gland, may obscure adrenal pathology and make PET measurements unreliable. Moreover, increased [^11^C]MTO uptake has been found in various liver lesions, such as adenoma, hepatocellular cancer and focal nodular hyperplasia (FNH), with the risk of false positive imaging results 8. Further, from a logistical point of view, the clinical availability of [^11^C]MTO PET/CT is restricted because of the short half-life of carbon-11 (T_1/2_= 20.4 min), which limits its use to PET centres with an in-house cyclotron and radiopharmacy 9-12.

Over and above the need to develop a radiotracer with better imaging properties to facilitate adrenocortical and retroperitoneal tumour imaging, alternative diagnostic procedures for PA are also needed. Current procedures lack sensitivity, and adrenal venous sampling is cumbersome, invasive and technically demanding, while frequently failing to produce conclusive results. Initial results in small groups of patients have indicated that diagnosis and lateralization of PA by [^11^C]MTO PET is feasible 4. A specific advantage would be to image expression of aldosterone synthase. However, the chemical structures of 11-β-hydroxylase are very similar, and MTO binds to both of these enzymes. This has been overcome by a few days of dexamethasone pre-treatment, leading to downregulation of 11β-hydroxylase, and the subsequent imaging shows the result of binding mostly to aldosterone synthase 4.

Consequently, it is necessary to develop a new PET tracer for adrenocortical imaging which, compared to [^11^C]MTO, has lower liver uptake and a longer half-life for better clinical availability.

**Figure 1:**
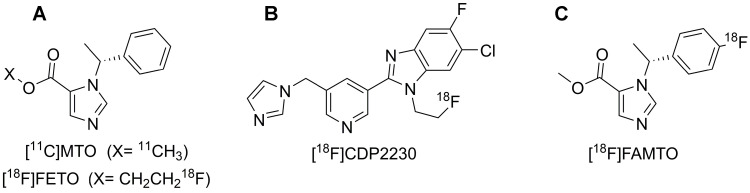
Displays the molecular structure of **A:** [^11^C]MTO & [^18^F]FETO, **B:** [^18^F]CDP2230 and **C:** [^18^F]FAMTO.

2-[^18^F]fluoroethyl-etomidate ([^18^F]FETO) (Fig. 1A) has already been used as an adrenocortical imaging agent. A first-in-man study demonstrated high 11β-hydroxylase selectivity 13 but [^18^F]FETO has not reached widespread clinical use, partial due to its two-stage radiosynthesis. In 2016, a promising new imaging agent, [^18^F]CDP2230 (Fig. 1B), was described for lateralization in PA. The tracer demonstrated selectivity for aldosterone synthase *in vitro* superior to that of [^11^C]MTO (Fig. 1A), but further studies are needed 14. Recently, promising preclinical *in vitro* and *in vivo* results for a fluorine-18 analogue of MTO, [^18^F]FAMTO (Fig. 1C), were published 15.

In a previous comparative study, where a panel of carbon-11 and fluorine-18 labelled metomidate and etomidate analogues was evaluated, para-chloro-2-[^18^F]fluoroethyl-etomidate, [^18^F]CETO - showed beneficial characteristics, superior to those of MTO, including low liver uptake.^16^ Based on these findings, we have established a GMP production method for para-chloro-2-[^18^F]fluoroethyl-etomidate, denoted [^18^F]CETO, and have further evaluated its binding properties and *in vivo* behaviour. For clinical adrenocortical imaging, [^18^F]FETO and [^11^C]MTO are currently available and were used for comparison with [^18^F]CETO.

## Materials and methods

### Chemistry

#### Radiosynthesis of [^18^F]CETO

**Figure 2:**
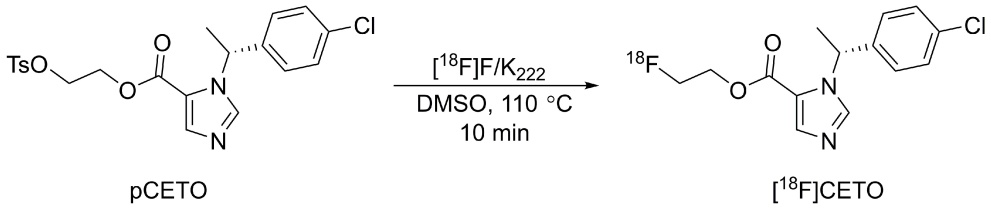
Radiosynthesis of [^18^F]CETO.

[^18^F]CETO was synthesized (Fig. 2), according to a slight modification of previously published procedures^16^, using a precursor obtained from Pharmasynt AS (Tartu, Estonia). Identification was done by NMR (^1^H and ^13^C) and HPLC, and the chemical purity was > 97% as determined by HPLC.

[^18^F]fluoride was trapped onto a QMA SPE cartridge and eluted with 900 µL of 100 mg K_222_ in 8 mL dry acetonitrile and 14 mg K_2_CO_3_ in 2 mL sterile water, and dried at 120 °C under vacuum. 2-3 mg of pCETO in 500 µL of DMSO was reacted with [^18^F]fluoride at 110 °C for 10 min. The reaction mixture was cooled and diluted with 2 mL water, after which [^18^F]CETO was purified by high-performance liquid chromatography (HPLC), (ACE-HL five, 250×10 mm) using 50% MeCN in a 50 mM ammonium formate buffer. Unreacted [^18^F]fluoride was separated prior to semi-preparative HPLC using an Alu N SPE cartridge. The solvents were removed by evaporation, and pure [^18^F]CETO was formulated in 7.2 mL of PBS (pH 7.4) containing 150 mg hydroxypropyl-β-cyclodextrin and 0.8 mL of sterile ethanol. Starting with 20-30 GBq of [^18^F]fluoride gave approximately 2-3 GBq of pure [^18^F]CETO (RCP >99%) with a synthesis time of around 90 min including QC. There were no traces of precursor and the molar activity was ~300 GBq/µmol at EOS.

For purposes of comparison, [^11^C]MTO and [^18^F]FETO were prepared by slightly modified procedures as previously described 11, 17.

### Frozen section autoradiography

Tissues from normal human adrenal glands, liver, kidney, spleen and small intestine were used along with samples of different types of adrenal tumours, aldosterone-producing adenomas (APA), cortisol-producing adenomas (CPA), ACC, and pheochromocytoma (PCC). Normal adrenal gland tissues and spleen tissues from non-human primates (NHPs) were also utilized.

Specimens for autoradiography were prepared from snap-frozen tissue, stored at -70 °C, and divided by a cryotome into 20 μm thick slices. The slices were placed on Superfrost^TM^ glass slides and stored at -20 °C until the autoradiographic examination. Slides were pre-incubated at room temperature for 20 min in a PBS buffer. Duplicate slides were incubated in 0.03 nmol/L [^18^F]CETO for 60 min, in the presence or absence of an excess of metomidate (0.45 µmol/L) to block the specific binding of [^18^F]CETO. After incubation, slides were washed for 3 × 3 min in cold PBS buffer, followed by rapid immersion in water. Slides were then dried for 10 min at 37 °C and exposed to Super Resolution Storage Phosphor Screens (PerkinElmer, Downers Grove, IL, USA) for >240 min and scanned in a Cyclone Plus Phosphor Imager (PerkinElmer, Model C431200, Downers Grove, IL, USA).

### Frozen section immunohistochemistry

The specimens for frozen section immunohistochemistry (IHC) were prepared from snap-frozen tissue, stored at -70 °C. They were partitioned with a cryotome into 6 μm thick slices. Subsequently, the specimens were placed on Superfrost^TM^ glass slides, incubated in acetone for 15 min and stored at -20° until IHC. Further details are described in the supplementary information.

### *In vitro* binding studies, [^18^F]CETO

Normal NHP adrenal tissue was weighed and homogenized in 20 volumes of ice-cold 0.32 M sucrose and stored in aliquots at -80 °C until use. For the saturation binding study, the homogenate was further diluted to 20 µg/mL in the assay. Triplicates of each ligand concentration in the range of 0.03-30 nmol/L [^18^F]CETO, with and without a competing ligand (1 µmol/L metomidate), were incubated at room temperature for 60 min. For the competition binding assay, 200 µg/mL of NHP adrenal gland tissue was used. Triplicate tubes, containing 10 nM [^18^F]CETO were incubated for 60 min at room temperature with three competing ligands, MTO, FETO and CETO, at concentrations between 0 and 30 nmol/L, respectively.

The incubations were terminated by filtration under vacuum through Whatman GF/C glass fibre filters, using a cell harvester (Brandel, Gaithersburg, MD). The filters were washed four times with 3 mL PBS buffer and transferred to scintillation tubes where the trapped radioactivity was measured in an automated gamma counter (Wizard 2480, PerkinElmer Downers Grove, IL, USA).

The saturation study was repeated twice with normal NHP adrenal gland tissue and the results were used to calculate the equilibrium dissociation constant, K_d_, and the total target density for the binding of [^18^F]CETO, B_max_. Values for IC_50_, B_max_ and K_d_ were estimated by GraphPad Prism 8.2, using non-linear regression of the binding curves.

To determine the *in vitro* kinetics for association and dissociation of [^18^F]CETO to normal NHP adrenal tissue, 20 µL of homogenate, corresponding to 40 µg of adrenal, was pipetted to a Poly-D-lysine coated cell dish (CELLCOAT™, Greiner Bio-One GmbH, Austria) and allowed to dry for 15 min. Subsequently, the dishes were placed in the inclined rotating holder of a LigandTracer™ yellow instrument (Ridgeview Instruments AB, Uppsala, Sweden) and [^18^F]CETO was added with concentrations ranging from 1 to 5 nM to measure the association rate. A higher concentration was added when the previous concentration had reached equilibrium. To measure the dissociation rate, the radioactive solution was replaced with buffer after the highest concentration reached equilibrium. Data were analyzed with TraceDrawer software (Ridgeview Instruments AB, Uppsala, Sweden) and the association rate constant (k_on_), dissociation rate constant (k_off_) and equilibrium dissociation constant (K_D_) were computed using a 1:1 kinetic binding model.

### Organ distribution

A biodistribution study for [^18^F]CETO was performed in thirty Sprague Dawley rats. A dose of 5.4±0.9 MBq [^18^F]CETO was injected as a bolus in the lateral tail vein, to un-sedated rats. Three rats of each gender were then euthanized by high concentrations of CO_2_, at five predetermined time points; 10, 30, 60, 120 and 240 min post injection (p.i.), respectively, and their organs were harvested and weighed. Radioactivity content in the organs was measured in a well-type NaI(Tl) scintillation counter, applying correction for dead-time and for decay. The organ radioactivity readings were decay-corrected to the time of injection, and results were expressed as standardized uptake values (SUV).

### *In vivo* imaging studies on rodents

#### General

Sevoflurane inhalation was used for the induction and maintenance of anaesthesia, and an intravenous catheter was placed in the lateral tail vein. After the investigations, the mice/rats were euthanized and their organs harvested and weighed, followed by measurement of radioactivity (as previously described). PET and MRI images were acquired on a Nanoscan PET-3TMRI scanner (Mediso Mediso, Medical Imaging Systems, Budapest, Hungary).

#### PET MRI with [^18^F]CETO in mice

Eight female C57BL/6 mice were positioned on the heated bed of the PET/MRI scanner and a dynamic PET acquisition of the abdomen was initiated immediately before injection of 1.6±1 MBq of [^18^F]CETO. Four of the mice were co-injected with metomidate (1 µmol/kg). The PET examination was continued for 60 min, and was followed by a 15 min MRI acquisition.

#### [^18^F]CETO PET-MRI in rats

Two male Sprague Dawley rats were positioned on the heated bed of the scanner and a dynamic PET acquisition of the abdomen was initiated immediately before injection of 4-5 MBq of [^18^F]CETO. Metomidate (1 µmol/kg) was co-injected in one of the rats as a blocker. The dynamic PET scan was continued for 60 min and was followed by a whole-body static scan, 3 beds, 5 min per bed position ~90 min post injection, and by MRI of the abdomen.

#### [^18^F]FETO PET-MRI in rats

PET acquisition was initiated and 4.0-4.8 MBq of [^18^F]FETO was intravenously administered. One of the rats was given metomidate (1 µmol/kg) I.V., for a blockage study. An initial dynamic PET scan of the abdomen was performed for 60 min, followed by a baseline whole body PET scan for 15 min.

### Non-human primate experiment

One female NHP, *Macaca fascicularis,* was obtained from the Astrid Fagræus Laboratory, Karolinska Institute, Comparative Medicine. The NHP was used to compare the [^18^F]CETO and [^11^C]MTO biodistribution and their uptake over time in adrenal and liver.

Ketamine (~10 mg/kg) was used as a sedative. Whilst being transported to the PET centre at Uppsala University Hospital, the NPH was weighed and maintained on iterated ketamine injections (15 mg/kg/h). Saturation levels were continuously measured through pulse oximetry, and oxygen administered as needed.

At the PET centre, the NPH was intubated under propofol-induced anaesthesia. General anaesthesia was maintained by sevoflurane inhalation (1.5 - 5.0%) and artificial ventilation. Two venous catheters were placed - one for blood sampling and one for tracer administration. Body temperature, heart rate, ECG, pCO_2_, pO_2_, SaO_2_ and blood pressure levels were monitored throughout the PET study. A General Electric Discovery MI PET/CT scanner (GE Healthcare, Milwaukee, USA) was used 18.

PET/CT positioning was determined by using a scout CT scan, and 6.5 MBq/kg of [^11^C]MTO was administered I.V. through the catheter. A baseline dynamic 90 min PET scan over the abdomen was started. After a sufficient waiting period, to allow for the radioactive decay of carbon-11, 4.2 MBq/kg of [^18^F]CETO was injected I.V. A 90 min baseline dynamic PET scan was started and was followed by a 15 min whole-body PET examination. Thereafter, a blockage study was performed by administering 0.5 mg/kg etomidate I.V. for 20 min. At the end of etomidate administration, corresponding to approximately 150 min after the baseline [^18^F]CETO injection, 19.3 MB/kg of [^18^F]CETO was administered I.V., followed by a 90 min dynamic PET scan. The amount of radioactivity used in the blocking study was nearly fivefold that used during the baseline PET examination, in order to render any remaining activity from the baseline scan negligible. During both [^18^F]CETO PET scans, discrete venous blood samples (~2 mL) were collected in heparinized tubes at 5, 10, 20, 40 and 80 min p.i. (3 mL at the last time point) of [^18^F]CETO to measure radioactivity in whole blood and in plasma and for metabolite analysis. PET images were reconstructed using an ordered-subsets maximisation (OSEM) algorithm including time-of-flight information and resolution recovery. Dynamic images were reconstructed into time frames with increasing durations.

After the imaging sessions, the NHP was wakened and de-intubated and, once stable, transported back to its living facility.

#### Radio metabolite analysis of [^18^F]CETO in NHP

The analysis of radio-labelled metabolites in one NHP was performed according to a previously described procedure, with slight modifications.^16^ Each blood sample was weighed, measured for radioactivity in the γ-counter and subsequently centrifuged at 3000 × g for 2 min at 4 °C (Beckman Allegra X 22R Centrifuge, Palo Alto, USA) to separate the plasma. The plasma was then weighed, measured for radioactivity and stored on ice. In the next step, 800 µL of plasma was added in a small separate vial followed by an equal amount of acetonitrile to precipitate proteins from the plasma, and centrifuged for 1 min at 16000 x g at 4 °C (Eppendorf 5415R Centrifuge, Eppendorf AG, Hamburg, Germany). The supernatant was filtered through a 0.2 μm nylon membrane (Corning Incorporated, Corning, NY, USA) by centrifugation at 16,000 × g at 4 °C for 1 min. An authentic CETO reference compound (5 μL, 1.0 mg/mL) was then added to the mixture to aid identification of the parent compound fraction during HPLC separation and fraction collection. The sample preparation recovery was determined by measuring the radioactivity of plasma, filters and pellets. A reverse phase (RP) HPLC method was used to separate the radiometabolites from the parent radiotracer [^18^F]CETO. Further details are described in the supplementary information.

#### Kinetic modelling in NHP

In the PET images, volumes of interest (VOIs) were drawn over representative subsets of aorta, adrenals, kidneys, liver, lungs, pancreas and spleen and transferred to all dynamic time frames to obtain time-activity curves (TACs), showing SUV as a function of time, after normalization to injected activity per body weight. Plasma TACs were determined by multiplying the aorta TAC with the mean plasma-to-whole blood ratio of the five analysed blood samples. Then, a metabolite-corrected input curve, representing intact tracer in plasma, was calculated by multiplying the plasma TAC with a sigmoid fit to the measured parent fraction data.

Adrenal time-activity curves were fitted using single-tissue as well as irreversible and reversible two-tissue compartment models. The optimal model to describe the data was determined using the Akaike information criterion 19. In addition, data were analysed using the Patlak and Logan graphical analysis methods, assuming irreversible and reversible kinetics, respectively. Additionally, whole-body net uptake rate parametric images were calculated using the Patlak method.

#### Ethics

The study was approved by the Local Ethics Committee (Dnr 2012-422) and the Animal Ethics Committee (Dnrs C11/15, 5.8.18-00211/2017 and 5.8.18-14982/2017). Tissues were obtained from the Uppsala Biobank. All patients provided informed consent. All *in vivo* experiments on mice, rats and non-human primates (NHPs), including the PET/CT and PET/MRI with [^18^F]CETO, [^18^F]FETO or [^11^C]MTO, were conducted in accordance with current guidelines set up by the Swedish Animal Welfare Agency.

## Results

### Autoradiography including immunohistochemistry

**Figure 3:**
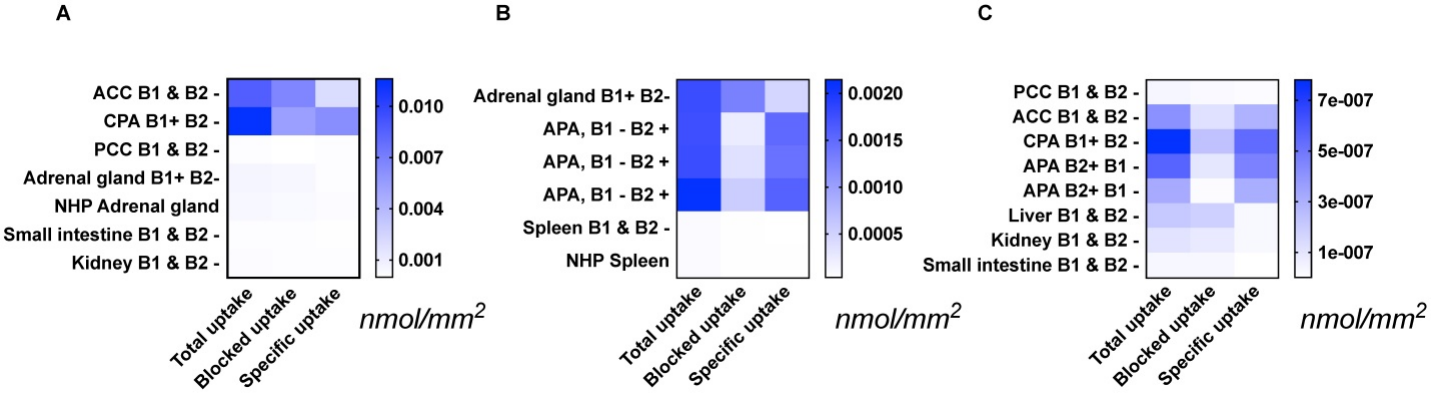
Tissue uptake of [^18^F]CETO (nmol/mm^2^) from the results of three separate autoradiography experiments A-C. The immunohistochemical expression of CYP11B1/-B2 is noted as B1/B2 positive (+) or negative (-). Abbrevations used in figure*:* ACC: adrenocortical carcinoma (**A, C**), APA: adrenocortical adenoma (**B, C**), CPA: cortisol-producing adenoma (**A, C**), NHP: non-human primate (**A**, **B**), PCC: pheochromocytoma (**A, C**). Note that the presented values can only be compared within each separate heat map:** A**, **B** or **C**.

Specific [^18^F]CETO uptake was elevated in APA, CPA and ACC (Figs. 3A, 3B). Specific uptake in human and NHP spleen was lower than in normal adrenal gland and adrenal tumours (Fig. 3B). Specific uptake in PCC, small intestine, kidney and liver were also lower than that in normal adrenal gland (Figs. 3A, 3C). [^18^F]CETO showed an approximately threefold higher uptake in APAs compared to that of the normal adrenal gland (Fig. 3B).

### *In vitro* binding studies

**Figure 4:**
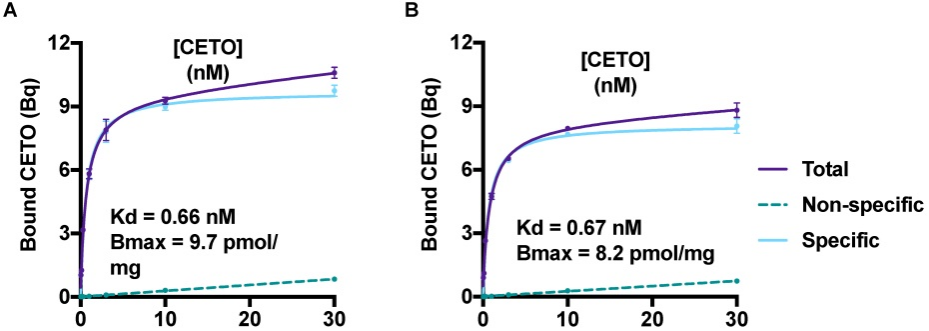
** A** &** B:** Saturation curves, from two separate experiments, generated B_max_ and K_d_ values for [^18^F]CETO. The graphs show the binding of [^18^F]CETO to CYP11B1 and CYP11B2 in NHP adrenal gland homogenate. Total binding (black line), non-specific binding (dark grey interrupted line) and specific binding (light grey), were obtained by non-linear regression analysis of the data in GraphPad Prism 8.2. Specific binding values were obtained by subtracting the non-specific binding from the total binding. The values represent the mean ± standard error of the experiment, which was performed using triplicates.

The saturation curves for [^18^F]CETO in the NHP adrenal gland homogenate are presented in Figure 4. The equilibrium dissociation constant, K_d_, for [^18^F]CETO in NHP adrenal gland was 0.66-0.67 nM, and the calculated B_max_ value 0.0082-0.0097 nmol/mg.

Results from the competition binding studies, in which the residual binding of [^18^F]CETO was determined in the presence of increasing concentrations of MTO, FETO and CETO, respectively, showed that the three ligands had similar *in vitro* affinities. MTO (IC_50_ = 2 nmol/L) displayed the highest affinity, followed by FETO (IC_50_ = 3 nmol/L) and CETO (IC_50_ = 5 nmol/L). The determination of the binding kinetics of [^18^F]CETO was well in accordance with the results obtained by the equilibrium saturation study. The K_d_ value derived from the rate constants was 0.59 nmol/L, and k_on_ was 5.9 × 105 M^-1^*s^-1^ and k_off_ was 3.5 × 10^-4^ s^-1^, which corresponds to a 33 min half-life for the complex.

### *Ex-vivo* biodistribution

**Figure 5:**
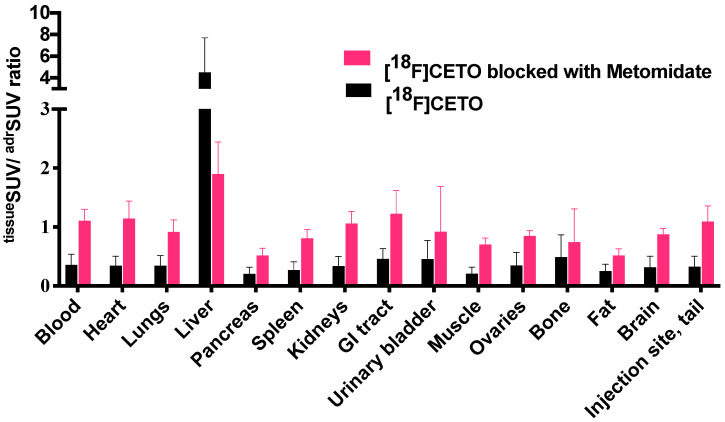
[^18^F]CETO *in vivo* biodistribution in female mice (N=4 per group), without (black bar) or with (grey bar) metomidate blocking, here expressed as ^tissue^SUV/^adrenal^SUV ratios, with individual ratios to be compared with 1 (^adrenal^SUV/^ adrenal^SUV).

**Figure 6:**
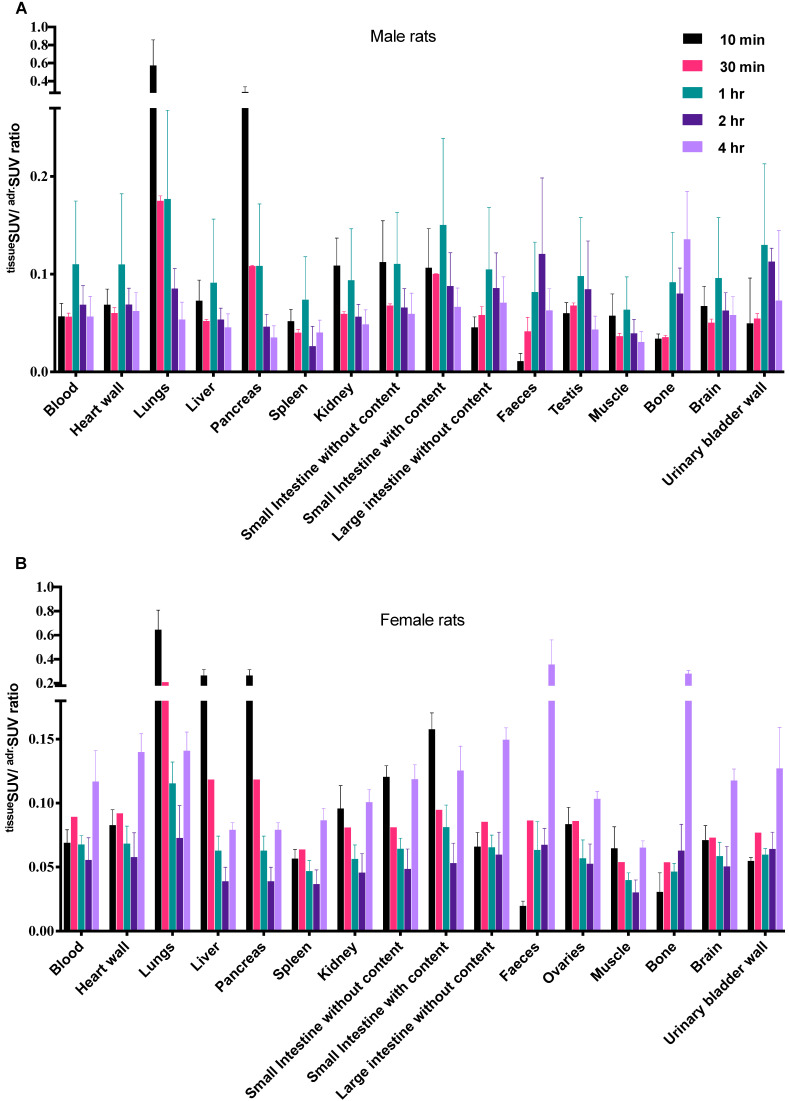
*Ex-vivo* biodistribution of [^18^F]CETO in rats.** A**: male rats, **B**: female rats; n= 3 per timepoint, with the exception of **B** at 30 min where n= 1.Data are expressed as ^tissue^SUV/^adrenal^SUV ratios, with individual ratios to be compared with 1 (^adrenal^SUV/^ adrenal^SUV).

The [^18^F]CETO biodistribution data in rats showed high accumulation in the adrenal glands with a peak uptake at 2 hr p.i. At 10 min p.i., the lungs and pancreas showed an initially high uptake followed by a rapid decline, whereas at other time points and in all other organs and tissues only low uptakes (approximately SUV 1) were detected. Exceptions were an accumulation in bone that increased slightly over time, suggesting tracer de-fluorination, which is a common feature for ^18^F-labelled molecules. Consistent with the expected tracer elimination via the kidneys, the uptake in the urinary bladder also gradually increased over time (Fig. 6). The *in vivo* biodistribution results of [^18^F]CETO in mice are presented in Figure 5.

### Animal imaging studies

**Figure 7:**
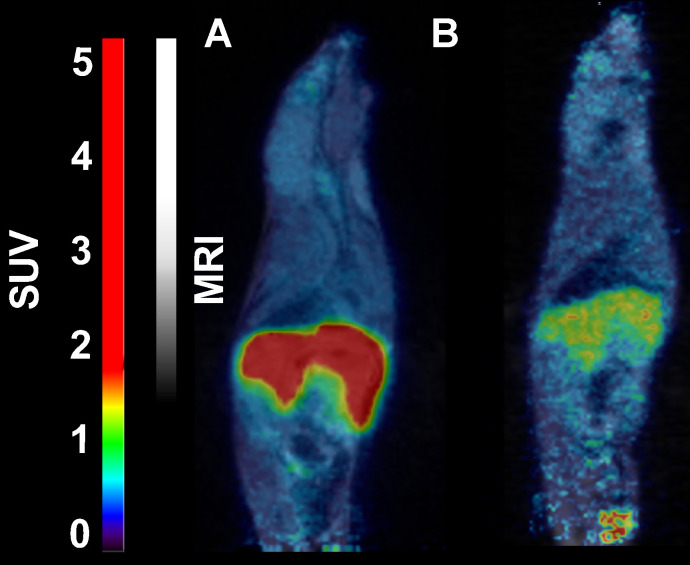
Representative sagittal PET-MR images of mice injected with [^18^F]CETO, SUV 30 min post injection. **A:** At baseline. **B:** After blockage with metomidate, 1μmol/kg.

In mice, [^18^F]CETO accumulated predominantly in the liver and in the adrenal glands, thus obfuscating the view of the adrenal glands (Fig. 7).

**Figure 8:**
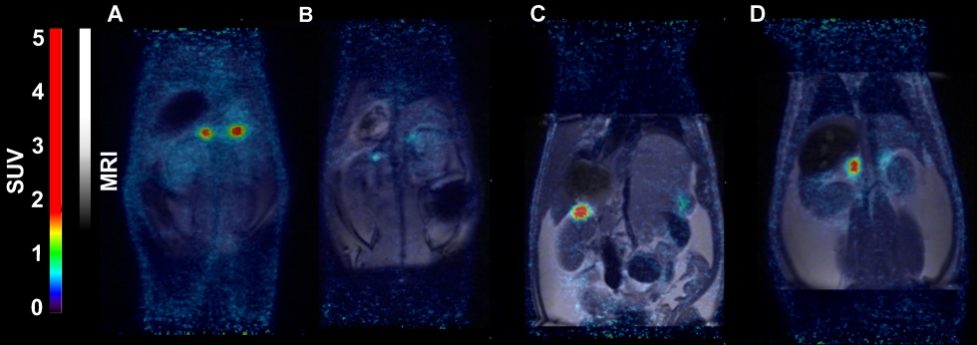
Representative coronal PET **(A)** and PET/MRI fusion **(B, C, D)** SUV images in rat 30 min post injection of [^18^F]CETO **(A, B)** and [^18^F]FETO **(C, D)**, without **(A, C)** and with (**B, D**) blockage with 1μmol/kg metomidate.

In rats, [^18^F]CETO uptake, by contrast, was concentrated mainly in the adrenal glands (Fig. 8A-B). Peak adrenal uptake was determined to 120 min p.i. in rats. The uptake of [^18^F]FETO in rats was also concentrated mainly in the adrenal glands. However, we were unable to block the uptake with metomidate (Fig. 8C-D). For both rats and NHPs, the elimination of [^18^F]CETO was mainly renal.

**Figure 9:**
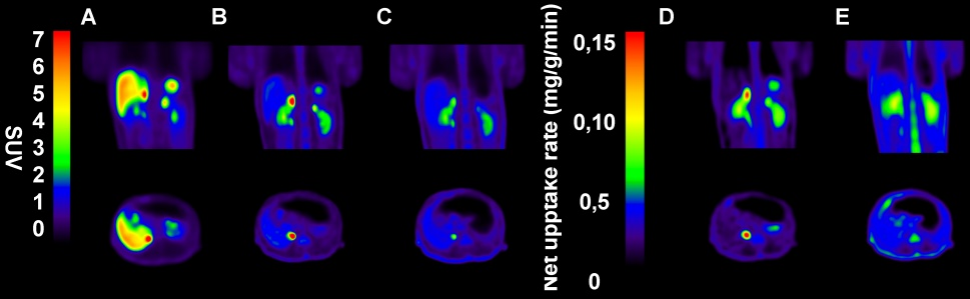
Representative sagittal (upper panel) and transverse (lower panel) PET images in NPH.** A-C:** SUV images 60-85 min post tracer injection. **D-E:** Net uptake rate (Patlak). **A:** [^11^C]MTO, **B:** [^18^F]CETO, **C:** [^18^F]CETO after blockage with etomidate 0.5 mg/kg, **D:** [^18^F]CETO, **E:** [^18^F]CETO after blockage with etomidate 0.5 mg/kg.

In NHPs, [^18^F]CETO-PET/CT showed a high specific uptake in the adrenal glands, and a low non-specific background uptake in the surrounding organs and tissues (including in the liver) (Fig. 9B).

**Figure 10:**
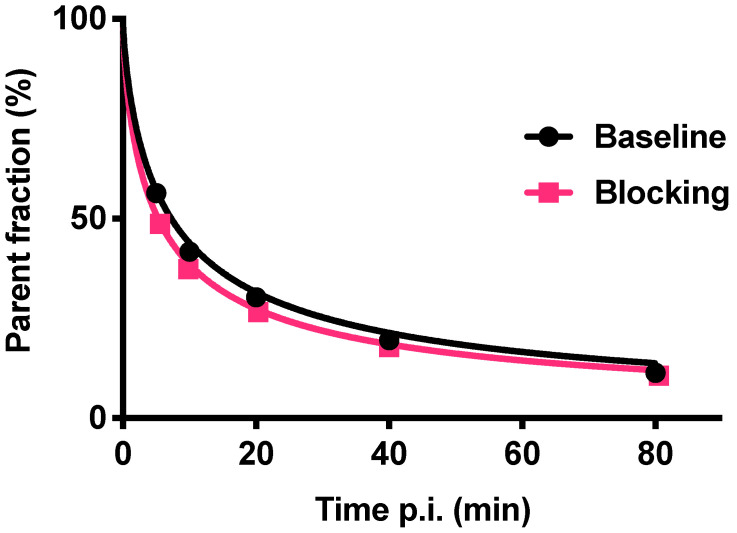
Fraction of intact [^18^F]CETO in venous plasma from NHP, at baseline (black line) and after etomidate blocking (pink line).

Radiometabolite analysis showed that 56% of [^18^F]CETO was intact at 5 min, which decreased to 11% at 80 min p.i. (Fig. 10). Radiometabolites were well separated from the radiotracer [^18^F]CETO in the chromatogram and all detected radiometabolites were more polar than the intact radiotracer from 5 to 80 min p.i. The recoveries of [^18^F]CETO during sample preparation and HPLC column injections were (84.6±1.3)% and (97.6±0.9)%, respectively.

**Figure 11:**
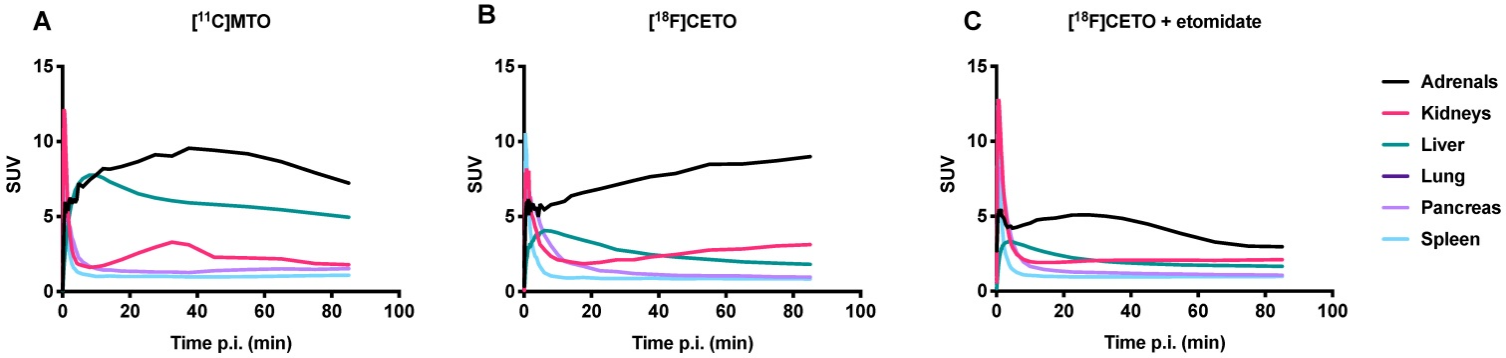
TACs showing the SUVmean over time in various NHP organs. **A:** the peak uptake of [^11^C]MTO in the adrenal glands was seen at approximately 40 min p.i. **B:** the peak uptake of [^18^F]CETO in the adrenal glands was still not reached at the end of scanning 85 min p.i. **C:** [^18^F]CETO after blocking with etomidate.

**Figure 12:**
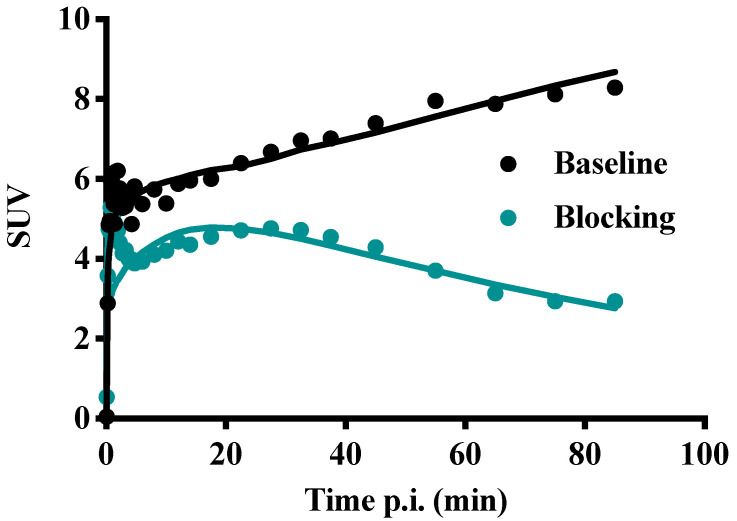
TACs (dots) and irreversible two-tissue compartment model fits for [^18^F]CETO in the adrenal glands at baseline (teal line) and after blocking with etomidate (black line).

Figure 11 shows TACs in relevant organs for [^11^C]MTO and [^18^F]CETO. In Figure 10A, the [^11^C]MTO uptake in the adrenal glands showed a negative slope at the end of the experiment. By contrast, in Figure 11B, the corresponding curves for the [^18^F]CETO uptake show that the accumulation in the adrenal glands was still increasing at the end of PET imaging. Kinetics of [^18^F]CETO in NHP at baseline could best be described by an irreversible two-tissue compartment model in all organs except the spleen, where a single-tissue comparison model was the best fit for the data. After blocking, the irreversible two-tissue compartment model was still preferred in all organs except the adrenal glands. In the adrenal glands, the rate constant k_3_ was reduced to zero, indicating complete blocking and leading to indistinguishable fits for the irreversible two-tissue and single-tissue compartment models. Figure 12 shows irreversible two-tissue compartment model fits of the adrenal gland TACS at baseline and after blocking (A).

No adverse effects were noted in the rats following [^18^F]CETO injection. Equally, the NHPs, which had an anticipated and seemingly normal recovery post-anaesthesia, displayed no adverse effects.

In NHPs, the highest [^18^F]CETO adrenal-to-liver ratio of >5 was reached 85 min p.i. and for [^11^C]MTO this was 1.5 at 40 minutes p.i.

## Discussion

In this study, the evaluation of [^18^F]CETO, as a new PET tracer for the clinical imaging and characterisation of adrenocortical tumours, showed specific binding with high affinity in the normal adrenal cortex as well as in human adrenocortical adenomas (APA, CPA) and adrenocortical carcinomas, both *in vitro* (in human) and *in vivo* (in rat and NHP) with low liver uptake in rat and NHP.

The initial *in vivo* studies, performed on mice, showed specific binding of [^18^F]CETO to the adrenal glands, but also showed a considerable unspecific uptake in the liver, making the right adrenal gland difficult to visualize. Consequently, the remaining experiments were performed in rat and NHP, both of which (particularly the NHP) exhibit enzymatic profiles generally more closely resembling humans. However, a re-evaluation of [^18^F]FETO had to be discontinued because in rats it was not possible to block the uptake *in vivo* using metomidate as the blocking substance (Figure 7D). Only one NHP experiment was performed, due to concerns regarding the animal welfare.

Recent findings have established that the expression of aldosterone synthase is heterogeneous, i.e. its expression is variable, depending for instance on the underlying molecular derangement, and that aldosterone-producing cell clusters (APCCs) in the zona glomerulosa of the adrenal cortex express high levels of aldosterone synthase 20, 21. Frozen section autoradiography performed with [^18^F]CETO, with or without metomidate as a blocking agent, showed that the binding was specific to adrenocortical adenomas (APA and CPA) and adrenocortical carcinomas but not to pheochromocytomas, which showed only unspecific background uptake. As expected, the accumulation displayed a heterogeneous pattern, varying between the different types of adrenocortical tumours (Figure 2).

Uptake in adrenocortical tissue exceeded that in the adjacent organs (liver, kidneys, small intestines, spleen). Furthermore, *in vitro* analyses of rat tissues following PET imaging showed high and specific uptake of [^18^F]CETO in the adrenal glands. Taken together, all [^18^F]CETO results, with or without metomidate as blocking agent, illustrated adrenocortical tissue-specific binding properties.

Results from the *in vitro* pharmacodynamic study indicated that [^18^F]CETO exhibits fast binding kinetics and a high affinity for adrenocortical tissue. The K_d_ value derived from the rate constants, 0.59 nmol/L, corresponds well to the obtained K_d_-values of 0.66-0.67 nm/L from the saturation study. However, the competition binding study showed that [^18^F]CETO does not evince a lower IC_50_ compared to [^11C^]MTO and [^18^F]FETO. The kinetic study showed a limited half-life of approximately 33 minutes for the [^18^F]CETO cell complex.

In NHPs and rats, [^18^F]CETO displayed a more favourable biodistribution profile than [^11^C]MTO with higher uptake of [^18^F]CETO in the adrenal glands and lower accumulation in the liver as compared to [^11^C]MTO. The adrenal glands (especially the right one) were thus easier to visualize with [^18^F]CETO. In NHPs, dynamic acquisition showed that the adrenal gland uptake of [^18^F]CETO continued to increase up to our end observation time point at 85 minutes, indicating an irreversible binding *in vivo*, or at least no signs of reversibility within the duration of the scan, contrary to the *in vitro* findings. This was supported by the finding that the irreversible two-tissue compartment model yielded the best fit of the data (Figure 11A-B). This was in contrast to the washout of tracer from surrounding organs, indicating that the theoretically optimal time point for [^18^F]CETO-PET/CT acquisition may be even later than 85 minutes p.i. in the clinical setting, although the present preclinical imaging findings cannot directly be translated to the human situation. However, besides having a higher adrenal-to-liver ratio than [^11^C]MTO , the notion of a later optimal scanning time creates possibilities for imaging of very small adrenocortical at a time point when the tumour-to-normal adrenal contrast may be higher. The reason for the difference between *in vitro* and *in vivo* kinetics is not clear. However, the high specificity in binding *in vivo*, as shown by the blockage studies, verify that *in vivo* kinetics are relevant and that data are not confounded by non-specific binding by radiolabeled metabolites.

[^18^F]CETO showed a bi-phasic metabolism with a fast first phase, approximately 10 min plasma half-life, followed by a second, slower phase. At the final observation time point 90 min p.i., [^18^F]CETO represented approximately 10% of radioactivity in plasma. Thus, the plasma half-life of [^18^F]CETO was sufficiently long to achieve high uptake in the adrenal cortex, and the elimination was fast enough to result in low background in non-target tissue, apart from the urinary bladder.

In summary, [^18^F]CETO shows promising characteristics with specific binding with high affinity in the normal adrenal cortex and, in rats and NHPs, very high adrenal uptake as well as low liver uptake, which facilitates depiction of the right adrenal gland. These results warrant further clinical assessment of [^18^F]CETO in order to improve adrenocortical PET/CT imaging in ACC and PA. The longer half-life of [^18^F]CETO, as compared to [^11^C]MTO, allows for distribution to PET centres with no in-house cyclotron.

## Supplementary Material

Supplementary materials.

## Abbreviations

ACC: adrenocortical carcinoma
APA: adrenocortical adenoma
ARR: aldosterone to renin ratio
AVS: adrenal vein sampling
APCC: aldosterone-producing cell cluster
BAH: bilateral adrenal hyperplasia
CPA: cortisol-producing adenoma
CT: computed tomography
CYP11B1: 1-ß-hydroxylase
CYP11B2: aldosterone synthase
GMP: Good Manufacturing Practice
MR: magnetic resonance tomography
NHP: non-human primate
OSEM: ordered subsets maximization
PA: primary aldosteronism
PCC: pheochromocytoma
PET: positron emission tomography
TAC: time-activity curve
VOIs: volumes of interest
[^18^F]CETO: para-chloro-2-[^18^F]fluoroethyletomidate
[^18^F]FETO: 2-[^18^F]fluoroethyletomidate
[^11^C]MTO: [^11^C]metomidate
