# Supplementary material for: Para-chloro-2-[18F]fluoroethyl-etomidate: A promising new PET radiotracer for adrenocortical imaging
Source: Int J Med Sci. 2021 Mar 21;18(10):2187–96. doi: 10.7150/ijms.51206 (PMC8040415; doi:10.7150/ijms.51206)
Supplement: Supplementary file 1 — Supplementary materials. [file ijmsv18p2187s1.pdf]

Frozen section immunohistochemistry

Slides were incubated, first in a PBS buffer and then in a 0.01% H202 solution in PBS to block endogenous peroxide production, for 15 min at room temperature. They were subsequently rinsed three times in a PBS buffer. Using a VECTASTAIN Elite ABC HRP Kit (Peroxidase, Standard), the slides were incubated in a humidified chamber with a drop of Avidin d for 15 min. They were then rinsed three times in PBS buffer both before and after re-incubation for 15 min, this time with a drop of biotin to reduce non-specific binding to tissues. The slides were then incubated with a drop of goat serum (for CYP11B1 clone 80-7, MABS502 primary antibody) or a drop of horse-serum (for CYP11B2 clone 41-17B, MABS1251 primary antibody) for 15 min. The drop of serum was drained of the slides by 45 μL of primary antibody. The CYP11B2 antibody was diluted 1:500 and the CYP11B1 antibody was diluted 1:200. The slides with CYP11B2 antibody were incubated for 120 min in room temperature, whereas the slides with CYP11B1 antibody were incubated over night at 4°C. They were then rinsed three times in a PBS buffer and incubated for 30 min with a drop of secondary antibody, diluted 1:200. Goat-anti-rat antibody was used for CYP11B1 and horse-anti-mouse antibody was used for CYP11B2. The slides were the rinsed 3 times in a PBS buffer, after which they were incubated for 30 min with a VECTASTAIN® ABC kit. The VECTASTAIN® ABC kit-solution was prepared 3 min before use. After incubation the slides were rinsed three times in a PBS buffer and incubated for 7 min with V Vector Laboratories SK-4100 DAB Substrate Kit, Brown (freshly made just before use: 0.05% DAB - 0.015% H_2O_2 in milli-Q water). The slides were rinsed in tap water and counterstained, by incubation for 2 min in hematoxylin, and then again rinsed in tap water. Tissue slides were dehydrated by immersion in three different alcohol solutions (75%, 95% and 99%) for 1 min per solution and then cleared in two xylen baths for 2 x 5 min. Coverslips were mounted using Pertex mounting medium, after which the slides were dried in room temperature.

Radio metabolite analysis of [^{18}F]CETO in NHP

Filtered plasma sample (1.5 mL) was diluted with milli Q water (0.5 mL) and 1.8 mL of sample was injected onto a semi-preparative HPLC RP column (ACE C18, 5 μm, 250 x 10 mm) equipped with a guard column (C18 SecurityGuard, 10 x 10 mm, Phenomenex). The column was eluted at a flow rate of 6 mL/min using the following gradient method: (50/50-90/10 B/A v/v, 0-10 min; 90/10 (B/A) v/v, 10-12 min; 90/10-50/50 B/A v/v, 12-15 min) with acetonitrile (B)-50 mM ammoniumformate pH 3.5 (A) as mobile phase. The sample was measured for radioactivity in the γ-counter before being analysed by HPLC and uninjected sample was also measured for radioactivity for calculating recovery of HPLC column injection. The radiometabolites and intact radiotracer were fractioned using a HPLC system (Gilson, Middleton, USA) equipped with a robotic liquid handler (ASPEC Gilson) monitoring at 254 nm with ultraviolet detector as well as with a radiodetector (Radiomatic 610TR, Packard, USA) for radiodetection. Six fractions were collected and radioactivity of all fractions were measured using γ-counter. The recovery of the HPLC column-injected radioactivity was calculated as: (sum of all fractions/injected sample - uninjected sample) x 100.
